# Cellular fate of intersex differentiation

**DOI:** 10.1038/s41419-021-03676-x

**Published:** 2021-04-12

**Authors:** Xin Wang, Fengling Lai, Dantong Shang, Yibin Cheng, Tian Lan, Hanhua Cheng, Rongjia Zhou

**Affiliations:** Hubei Key Laboratory of Cell Homeostasis, College of Life Sciences, Renmin Hospital of Wuhan University, Wuhan, China

**Keywords:** Germline development, Experimental models of disease

## Abstract

Infertile ovotestis (mixture of ovary and testis) often occurs in intersex individuals under certain pathological and physiological conditions. However, how ovotestis is formed remains largely unknown. Here, we report the first comprehensive single-cell developmental atlas of the model ovotestis. We provide an overview of cell identities and a roadmap of germline, niche, and stem cell development in ovotestis by cell lineage reconstruction and a uniform manifold approximation and projection. We identify common progenitors of germline stem cells with two states, which reveal their bipotential nature to differentiate into both spermatogonial stem cells and female germline stem cells. Moreover, we found that ovotestis infertility was caused by degradation of female germline cells via liquid–liquid phase separation of the proteasomes in the nucleus, and impaired histone-to-protamine replacement in spermatid differentiation. Notably, signaling pathways in gonadal niche cells and their interaction with germlines synergistically determined distinct cell fate of both male and female germlines. Overall, we reveal a cellular fate map of germline and niche cell development that shapes cell differentiation direction of ovotestis, and provide novel insights into ovotestis development.

## Introduction

Why and how sex is formed and maintained in two sexes, male and female, in most living organisms, have remained key questions in biology for over a century. Sexual reproduction exists in nearly all animals. In general, sex, as such a fundamental aspect of life, is nearly divided into male and female. However, the phenotype can be blurred in humans and other animals. Gynandromorph and intersex can happen in the same individual owing to, for example, genetic aberrations and/or developmental defects, in which the sex identity of cells is mixed together. In humans, patients with disorders of sex development (DSD) are often considered an ambiguity of phenotypic sex. DSDs are congenital conditions in which the development of chromosomal, gonadal, or anatomical sex is atypical^1^. Worldwide, genital ambiguity occurs in one in 2000–4500 in humans^2^; however, only approximately one-fifth of the DSDs can be diagnosed by molecular approaches^3^. Patients with DSDs are not only infertile, but also suffer from many health problems, including other malformations, developmental delay, intellectual impairment, and mental and psychosocial problems^1^. Understanding of sexual determination and differentiation mechanisms will benefit both diagnostic testing and medical therapy of DSDs.

In mammals, *SRY* on the Y chromosome is a dominant gene for male development^4^. In the embryonic gonads of males, SRY, together with NR5A1, upregulates *SOX9* expression via its enhancer^5,6^; thus, a developmental pathway for testis formation is initiated. Otherwise, in the absence of *SRY*, ovary development will start via several female pathways. RSPO1/WNT signaling is a major pathway for ovary development, which includes RSPO1, WNT4B, CTNNB1 and GSK3A/B^7,8^. Mutations in these sex-determining genes and the dysregulation of relevant pathways will lead to DSDs, including 46,XY complete or partial gonadal dysgenesis, 46,XX testicular DSD, and 46,XX ovotesticular DSD. For example, mutations in the conserved HMG-box and both 5′ and 3′ regions of sex-determining gene *SRY* caused XY gonadal dysgenesis/sex reversal, in which the ovarian structure was often in a degenerate state^9–12^. Mutations of *WT1*, *NR5A1*, and *FOG2* caused 46,XX testicular/ovotesticular DSDs^13–15^. A missense mutation in the *MAP3K1* gene, which reduced SOX9 expression of the male pathway and increased CTNNB1 activity in the female pathway, led to 46,XY complete gonadal dysgenesis^16^. Deletions/point mutations of *NR2F2* led to 46,XX ovotesticular DSD^17,18^. Gene duplications in sex-determining pathways can also lead to ovotesticular DSD. For example, a duplication of 1114 kb in the region of 17q24.3 containing *SOX9* was associated with *SRY* negative 46,XX ovotesticular DSD^19^, and duplications of *SOX3* also affected gonad development and resulted in 46,XX ovotesticular DSD in *SRY*-negative patients^20,21^. In addition, gonadal dysgenesis had a high risk for germ cell cancer formation^22^. Notably, mosaic karyotypes with three cell types, 46,X,dic(X;Y)(p22.33;p11.32)/45,X/45, and dic(X;Y)(p22.33;p11.32), were associated with ovotesticular DSD^23^, indicating that gene position/epigenetic effects could be contributed to genital ambiguity. Indeed, histone lysine acetyl-transferases were involved in epigenetic control of the *SRY* promoter^24^. Ovotesticular development is involved in, at least, two types of cell divisions (mitosis and meiosis) and two types of gametogenesis processes (spermatogenesis and oogenesis) within an ovotestis organ. Together, these data indicated that ovotesticular formation was a very complicated pathological process involved in multiple molecular and cellular events; however, the underlying etiology and molecular mechanisms are largely unknown.

Gonadal dysgenesis and ovotestis phenotype are often prevalent in animals, including dogs, cats, birds, fishes, amphibians, and reptiles^25–29^. In some animals, ovotestis formation is a physiological process, not a pathological condition, for example, in the teleost *Monopterus albus*, a new model species for evolution, genetics and development^26^. It begins life as a female, but transforms to a male through an intersex stage. The ovotestis model provides an ideal avenue to get insight into how the ovotestis is formed, although the underlying mechanisms have remained elusive since the 1940’s^30,31^. However, single-cell RNA sequencing (scRNA-seq) technology provides a new possibility to decipher the developmental events in the ovotestis at single-cell resolution. It shows a unique advantage in defining cellular identity, and tracing the cell state and fate in a whole organ^32–34^. Here, we performed extensive scRNA-seq characterization of the ovotestis using the 10x Genomics and related analysis applications. We first performed nonlinear dimensionality-reduction analysis, a uniform manifold approximation and projection (UMAP)^35^, on sequenced datasets after quality control, which yielded the first comprehensive single-cell transcriptional cell atlas of major cell types in the ovotestis, including germline, niche, and stem cells. Our study uncovered developmental changes in the lineages of these cell types. We identified common progenitors of germline stem cells with two states, revealing their bipotential nature to give rise to both spermatogonial stem cells (SSC) and female germline stem cells (FGSC), and two developmental trajectories of five subtypes of germline stem cells. Moreover, we revealed that ovotestis infertility was caused by developmental defects in both oogenesis and spermatogenesis processes, through nuclear phase separation of the proteasomes for degradation of female germline cells, and impaired histone-to-protamine replacement in spermatid differentiation. We also delineated signaling pathways in somatic niche cells and their interactive roles with germline cells in determining the cell fates of both testis and ovary differentiation. Together, our results provide a roadmap of germline, niche, and stem cell development in ovotestis, and reveal the molecular and cellular mechanisms underlying ovotestis formation in intersex.

## Results

### Two developmental trajectories of five subtypes of germline stem cells

**Fig. 1:**
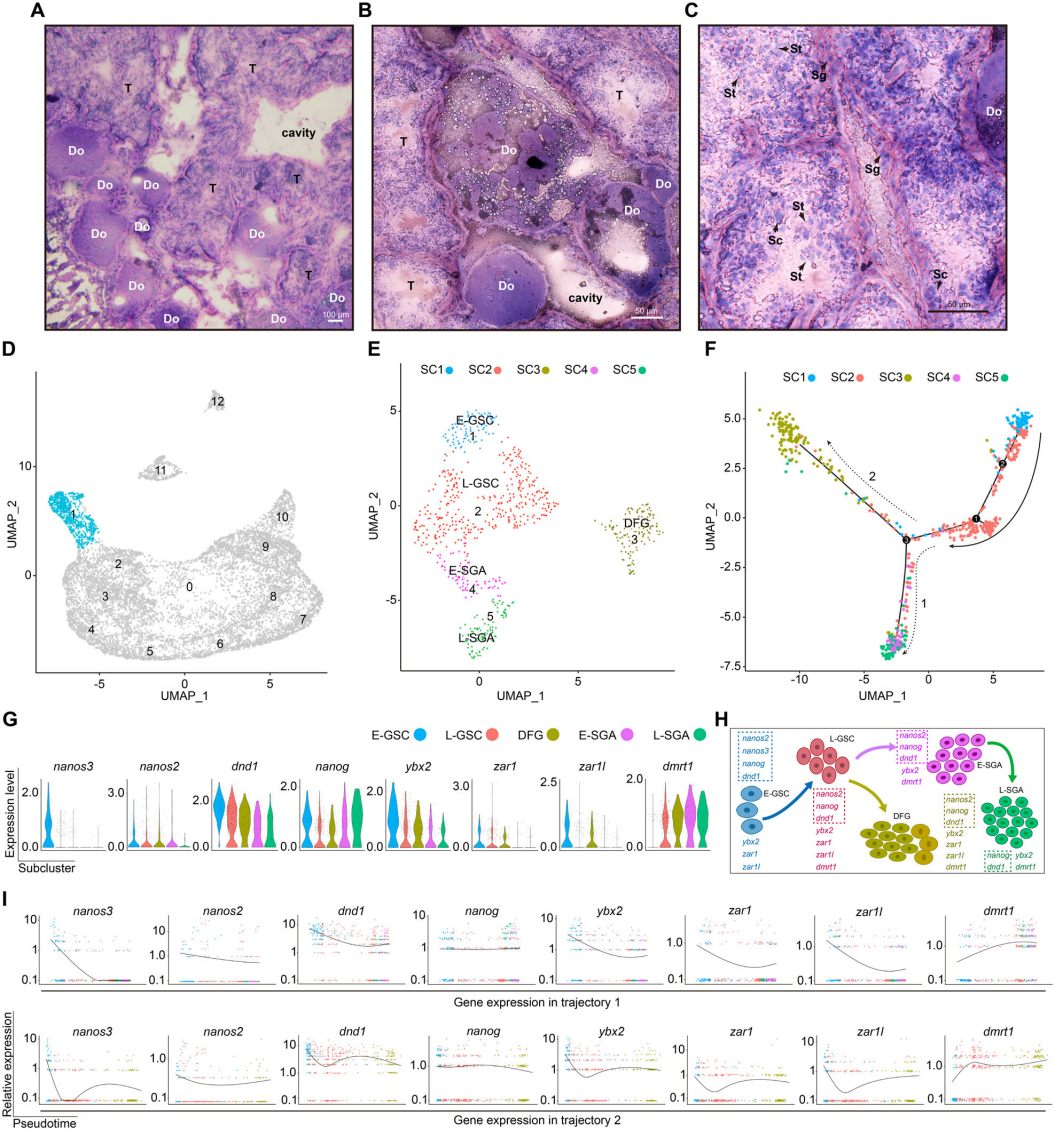
Identification of germline stem cells in ovotestis. **A** Ovotestis section stained by hematoxylin and eosin. DO degrading follicles, T testicular tissue. Scale bar, 100 µm. **B** Degrading follicles with various phenotypes in ovotestis. Scale bar, 50 µm. **C** Various spermatogenic cells, but lack of elongate spermatids in ovotestis. Sg spermatogonia, Sc spermatocytes, St round spermatids. Scale bar, 50 µm. **D** UMAP map showing cells in cluster 1 (blue). **E** Focused analysis of the cells in cluster 1 by UMAP clustering showed five subclusters: early-GSC (E-GSC), late-GSC (L-GSC), early-spermatogonia A (E-SGA), late-spermatogonia A (L-SGA), and degrading female germline cells (DFG). **F** Pseudotime trajectory analysis of five subclusters by Monocle revealed two differentiation trajectories (1 and 2). **G** Violin plots indicating expression levels of representative genes in these subclusters. **H** Schematic representation of stem cell lineage differentiation from E-GSC, via L-GSC, to L-SGA or to female germline stem cells. Stem cell factors are circled by dot lines. **I** Expression patterns of representative genes along the pseudotime axis. Top panels indicate pseudotime trajectory 1, and bottom panels show pseudotime trajectory 2.

Histologically, the ovotestis had atretic and degraded follicles (Fig. 1A, B), and spermatogonia, spermatocytes, and round spermatids (Fig. 1C). To explore the differentiation potential of ovotestis, we first sought to identify cell types in ovotestis using the single-cell RNA sequencing technology. We collected ovotestis samples from three intersex individuals and dissociated them into single cells for scRNA-seq. The sequencing depth was over 47,000 reads for each cell (Table S1), median detected genes were 3126 per cell, and median UMIs (unique molecular identifiers) were 9259 (Table S2). After discarding poor-quality cells, nearly 10,000 cells remained for clustering and typing. To identify cell clusters, we employed a nonlinear dimensionality-reduction technique, UMAP. Together with known marker genes and annotations (Table S3), 13 cell clusters were identified, including germline stem cells, primordial oocytes, spermatogonia, spermatocytes, round spermatids, and somatic niche cells (Sertoli and Leydig cells) (Fig. S1A). UMAP maps showed that each cluster was distinguished from other clusters with marker genes, for example, *nanos2* and *nanos3* in germline stem cells, *kif20a* and *kmt5a* in spermatogonia B, *spo11* and *meiob* in spermatocytes, *izumo1* and *spaca6* in round spermatids, *amh* and *wt1b* in Sertoli cells, and *cyp17a1* and *star* in Leydig cells (Fig. S1B).

As germline stem cells (GSC) were in cluster 1 (Fig. S1A, B), we next performed re-clustering analysis on the cluster to identify cell subtypes or states, which revealed the existence of five distinct subclusters (Fig. 1D, E). Of these, four subclusters (1, 2, 4, and 5) represented four states of germline stem cells, early-GSC (E-GSC), late-GSC (L-GSC), early-spermatogonia A (E-SGA), and late-spermatogonia A (L-SGA), while subcluster 3 was a group of female germline cells including female germline stem cells (FGSC). Pseudotime analysis revealed two developmental trajectories of these five subclusters; trajectory 1 transitioned from L-GSC to L-SGA for spermatogenesis, whereas trajectory 2 showed a distinct fate from L-GSC to female FGSC for oogenesis (Fig. 1F–H). Thus, E-GSC and L-GSC were two differentiation states of common progenitors of germline stem cells. E-GSC cells were primordial germline stem cells and L-GSC cells were bipotential to differentiate into either male (spermatogonia A) or female germline stem cells. Subcluster 3 contains FGSC as indicated by pluripotency markers *nanog2, nanog* and *dnd1*, in addition to *zar1 and zar1l*, key genes for oogenesis^36^ (Fig. 1G–I and Fig. S1A, B). These data suggested that L-GSC-to-E-SGA or L-GSC-to-FGSC transition potential existed in ovotestis. In line with this fact, upregulated genes were enriched in these two transitioning processes (Fig. S2A).

The expression patterns of key PGC markers^37,38^, including *nanos2, nanos3*, and *dnd1*, along with pluripotency marker *nanog* in E-GSC, confirmed the pluripotency of E-GSC cells. *nanos3* was expressed slightly higher in E-GSC than in L-GSC, indicating the relatively higher stemness of E-GSC in comparison with L-GSC (Fig. 1G–I). Pseudotime trajectory and expression of known cell state markers also indicated that E-SGA and L-SGA were two states of the type A spermatogonia, with high expression of *dmrt1*, but scarce expression of *zar1 and zar1l* (Fig. 1F–I). In mammals, *Dmrt1* was also expressed in the type A spermatogonia^39^. L-SGA expressed pluripotency markers *nanog* and *dnd1*, whereas spermatogonia B in cluster 2 did not express these two markers, but *kif20a* and *kmt5a*, important genes for cell division^40^ (Fig. S1B), indicating a transition from L-SGA to spermatogonia B in subsequent development. Notably, genes for DNA synthesis and mitosis were highly expressed in the germline stem cells, in particular, in type A spermatogonia, indicating that these germline stem cells were actively divided (Fig. S2B, C).

### Nuclear phase separation of the proteasomes promotes degradation of female germline cells

Considering functional loss of oogenesis, we explored the female germline cell fate in subcluster 3 (Fig. S3A). Apoptosis is a well-known mechanism for cell death. We indeed detected upregulation of pro-apoptosis gene expression, including *gadd45a, bax*, and *casp3a*, in subcluster 3 (Fig. S3B). We further investigated whether autophagy genes are involved in the process. All these genes in autophagy pathway, along with key genes for autophagosome induction (*atg13*, *ulk2*, *atg5*, *becn1*, *atg2b* and *pi3kr4*), elongation (*lc3a*, *lc3b*, *atg4b*, and *wipi2*), and fusion with lysosome (*tecpr1b* and *epg5*), were upregulated in subcluster 3 (Fig. S3C). Moreover, we found that *esr2b* and *atg13* were upregulated in subcluster 3 simultaneously (Fig. S3D). Functional tests showed that Esr2b binding to the *atg13* promoter was essential for activation of *atg13* transcription (Fig. S3E–H). Thus, autophagy was involved in female germline cell degradation in subcluster 3 (Fig. S3I).

**Fig. 2:**
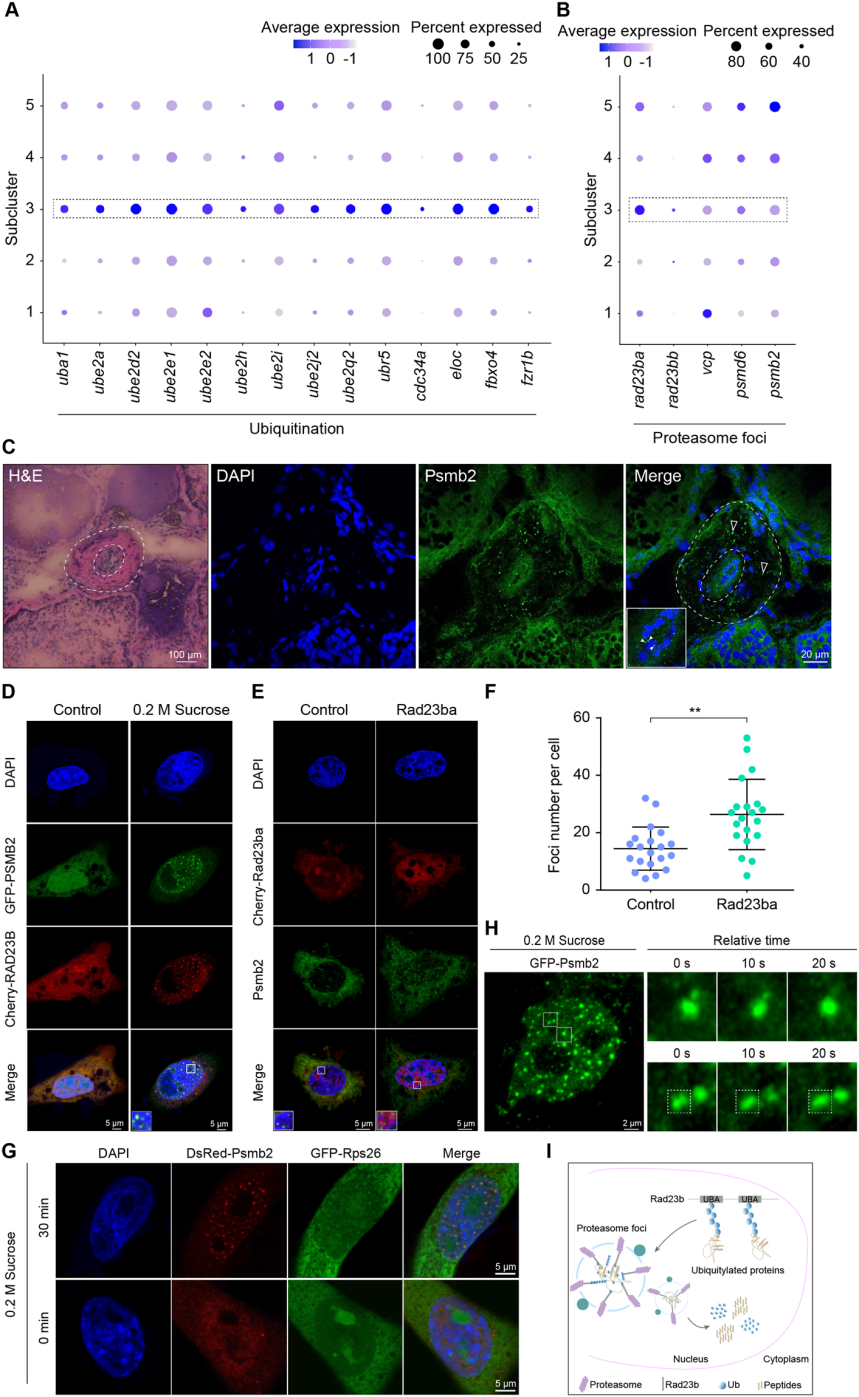
Nuclear phase separation of the proteasome for degradation of female germline cells. **A** Dotplot showing the expression of ubiquitination associated genes among subclusters. **B** Expression levels of key genes in proteasome foci formation among subclusters. **C** Immunofluorescence analysis of proteasome foci using Psmb2 antibody in ovotestis (secondary antibody, FITC-conjugated immunoPure goat anti-rabbit IgG(H + L)). Proteasome foci were located in the nuclei of germ cells (white solid arrowheads) and in the cytoplasm of supporting cells (white hollow arrowheads). The nuclei were stained with DAPI. The outer dotted circle indicates the degrading region and the inner dotted circle shows the germinal cradle. Scale bar: H&E, 100 µm; immunofluorescence, 20 µm. **D** Formation of proteasome foci in the nuclei of CHO cells. The cells were treated with 50 µM MG-132 for 1 h and then stimulated with 0.2 M sucrose for 30 min. PSMB2 (green) and RAD23B (red) were colocalized in the nuclei. The nuclei were stained by DAPI. The enlarged images originated from the regions with white square. Scale bar, 5 µm. **E** Overexpression of Rad23ba promotes the formation of proteasome foci. Endogenous proteasome foci were detected by PSMB2 antibody in CHO cells. PSMB2 (green) and Rad23ba (red) were colocalized in the nuclei. The nuclei were stained with DAPI. The cells were treated with 50 µM MG-132 for 1 h and then stimulated with 0.2 M sucrose for 30 min. The enlarged images originated from the regions with white square. Scale bar, 5 µm. **F** Statistical analysis showing the proteasome foci number per cell. Twenty cells were counted for each group. Data were mean ± SD, **P < 0.01. **G** Co-localization of Psmb2 (red) and Rps26 (green) in nucleus. The cells were stimulated with 0.2 M sucrose for 30 min. The nuclei were stained by DAPI. Scale bar, 5 µm. **H** Proteasome foci fusion in living CHO cells expressing GFP-Psmb2. The cells were treated with 0.2 M sucrose. Right panel, enlarged time-course views of the square in the left panel. Scale bar, 2 μm. **I** The diagram indicating the nuclear phase separation of proteasome and ubiquitylated protein degradation. The proteasome foci consist of Rad23b, polyubiquitin chain proteins, and proteasome.

Importantly, the ubiquitination pathway was enriched in subcluster 3 by KEGG analysis of differential expression genes, and most of the ubiquitination pathway genes were obviously upregulated, including ubiquitin-conjugating enzymes *ube1, ube2*, and *ube3* (Fig. 2A). This result suggested an importance of ubiquitination-mediated degradation of the female germ cells, because these enzymes catalyzed ubiquitin conjugation to mark cellular proteins for degradation^41^. To obtain further functional insights, we analyzed expression patterns of key genes involved in the nuclear proteasome foci, and found upregulation of key regulator *rad23b*, including *rad23ba* and *rad23bb* in subcluster 3 (Fig. 2B). Rad23 regulated proteasome foci formation by phase separation in the nucleoplasm^42^. Under physiological and pathological types of stress, formation of proteasome foci in the nucleus was a new mechanism for proteasome degradation of ribosomal proteins^42^, and loss-of-function mutations in most ribosomal proteins were lethal for cells^43^. Thus, the ubiquitination-dependent proteasome pathway in the nucleus probably mediated degradation of the female germ cells. To verify this, we performed immunofluorescence analysis of Psmb2, a key component of the foci, in ovotestis. Proteasome foci were detected in the germinal cradle (early-stage female germ cells and surrounding supporting cells) in ovotestis, particularly in the nuclei of germ cells and in the cytoplasm of supporting cells (Fig. 2C). Furthermore, proteasome foci were formed in the nuclei of ovary cells (CHO) under hyperosmotic stress (0.2 M sucrose) (Fig. 2D). Notably, overexpression of Rad23ba in CHO cells can promote formation of the foci indicated by endogenous PSMB2, and number of the foci in the nuclei was increased obviously in comparison with the control group (Fig. 2E, F). Considering the essential roles of ribosomal proteins for cell survival, we tested a set of ribosomal proteins and found Rps26 and Rps2 were co-localized with Psmb2 in the nuclei upon stimulation with 0.2 M sucrose for 30 min (Fig. 2G and Fig. S3J), indicating that Rps26 and Rps2 were degraded substrates in the proteasomes. Further time-lapse imaging showed that the proteasome foci with Psmb2 can fuse together, suggesting a nature of liquid droplets of the foci in the living cells (Fig. 2H and Supplementary Videos 1 and 2). Overall, these data suggested that nuclear proteasomes promoted degradation of female germline cells by ubiquitination-dependent phase separation (Fig. 2I).

### Signaling pathways in gonadal niche cells reveal distinct fate between oogenesis and spermatogenesis

**Fig. 3:**
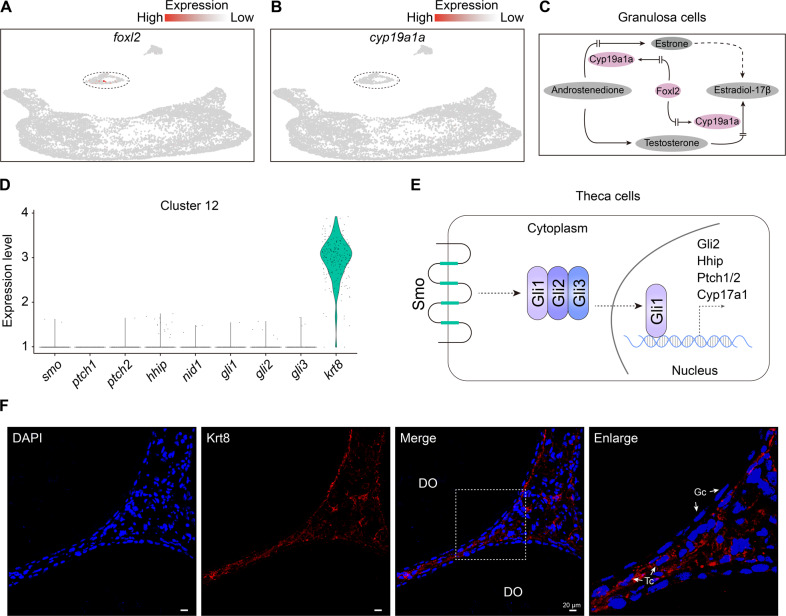
Loss of signaling pathways in the niche cells of ovary. **A** UMAP maps indicating scarce expression of *foxl2* in cluster 11. **B** Scarce expression of *cyp19a1a* in clusters 11. **C** Foxl2-Cyp19a1a pathway in female steroid biosynthesis process. **D** Relative expressions of hedgehog signaling pathway genes (*ptch1/2, smo, hhip, gli1*, *gli2*, and *gli3*), *nid1* (a gene encoding basal lamina-specific adhesive protein), and *krt8*. **E** Diagram indicating hedgehog signaling pathway in the theca cells. **F** Immunofluorescence analysis using Krt8 antibody in ovotestis (second antibody, TRITC-conjugated ImmunoPure goat anti-rabbit IgG (H + L)). The nuclei are stained with DAPI. Positive signals are located in Theca cells (arrows). The enlarged images on the right panel originated from the dotted line square on the left panel. DO degrading oocyte, Gc Granulosa cells, Tc Theca cells. Scale bar: 20 µm.

Considering key roles of the gonadal niche for germline cell development, we investigated signaling pathway activities for oogenesis. UMAP analysis showed that marker genes of granulosa cells were scarcely expressed, for example, faint expression of *foxl2* was detected in cluster 11 (Fig. 3A). There was also only 10.5% of Foxl2^+^ cells in the cluster 11, of which, ~6% were co-expressed with Sertoli markers, TGFβ (transforming growth factor beta) signaling pathway genes. Moreover, the *foxl2* downstream factor *cyp19a1a* was also scarcely detected (Fig. 3B), indicating loss of biosynthesis pathway of female sex hormone in the ovotestis (Fig. 3C). Hedgehog signaling pathway was essential for follicle development^44^. Key genes in the hedgehog signaling pathway in theca cells, including *ptch1/2*, *smo*, *hhip*, *gli1*, *gli2*, and *gli3*, were scarcely detected (Fig. 3D), indicating loss of hedgehog signaling pathway in ovotestis (Fig. 3E). In addition, expression of *nid1*, a basal lamina-specific adhesive protein in theca cells, was also very low (Fig. 3D), which was important for production of basal membranes of follicles^45^. Nevertheless, immunofluorescence analysis showed that theca cells can be detected by Krt8, which was expressed in cluster12 (Fig. 3D, F). However, Krt8 was not expressed in granulosa cells in the degraded follicles (Fig. 3F). These data suggested that the gonadal niche for oogenesis was functionally lost, although granulosa cells and theca cells co-existed in ovotestis.

**Fig. 4:**
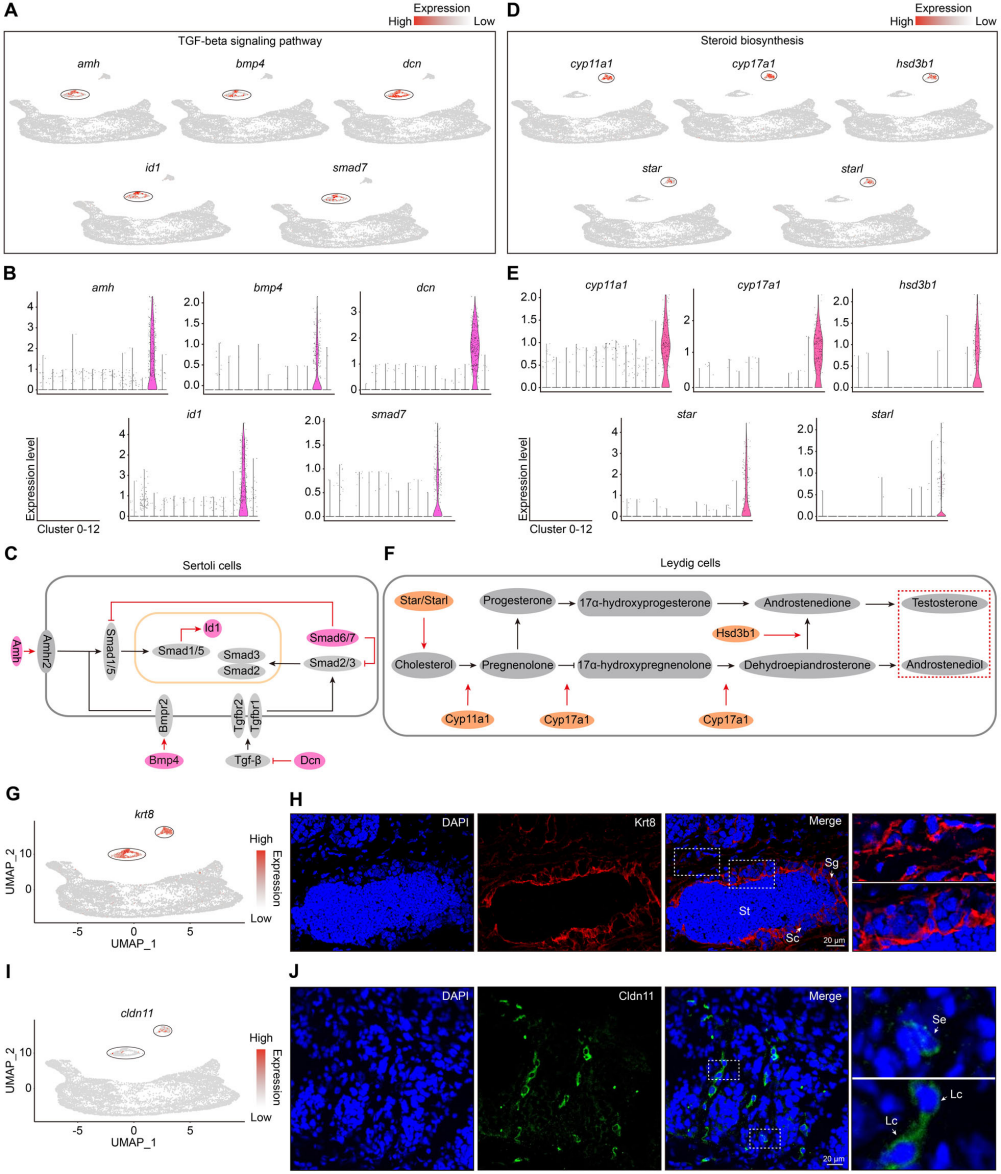
Signaling pathways in the niche cells of testis. **A** Key genes and expression patterns in TGFβ signaling pathway in Sertoli cells in cluster 11. Cluster-specific expression is indicated by black circles. **B** Violin plots showing expression levels of TGFβ signaling pathway genes. **C** TGFβ signaling pathway in Sertoli cells, highlighting the key genes in red ovals. **D** Key genes and expression patterns in steroid biosynthesis process in cluster 12. Cluster-specific expression is shown in black circles. **E** Violin plots showing expression levels of steroid biosynthesis genes. **F** Male steroid biosynthesis process in Leydig cells, highlighting the key genes in orange ovals. **G** UMAP map of *krt8* showing expression in both cluster 11 and 12, indicated by black circles. **H** Immunofluorescence analysis using Krt8 antibody in ovotestis (second antibody, TRITC-conjugated immunoPure goat anti-rabbit IgG(H + L)). The nuclei were stained with DAPI. Positive signals were located in Sertoli and Leydig cells (arrows). The enlarged images on the right panel originated from the dotted squares on the left panel. Sg spermatogonia, Sc spermatocytes, St round spermatids, Lc Leydig cells, Se Sertoli cells. Scale bar: 20 µm. **I** UMAP map of *cldn11* showing expression in both cluster 11 and 12, indicated by black circles. **J** Immunofluorescence analysis using Cldn11 antibody in ovotestis (second antibody, FITC-conjugated immunoPure goat anti-rabbit IgG(H + L)). The nuclei were stained with DAPI. Positive signals were located in Sertoli and Leydig cells (arrows). The enlarged images on the right panel originated from the dotted squares on the left panel. Lc Leydig cells, Se Sertoli cells. Scale bar: 20 µm.

As gonadal niche is essential for spermatogenesis, we then addressed signaling pathway activities in Sertoli cells and Leydig cells in ovotestis. KEGG pathway analysis by g:profiler^46^ showed that TGFβ family genes were enriched in Sertoli cells, including *amh, bmp4, smad7, dcn*, and *id1*. UMAP plots were built for Sertoli cells integrated by expression levels of TGFβ pathway genes (Fig. 4A, B). Key factors, including Amh, Bmp4, Dcn, Smad7, and their target Id1, in the pathway, were active in Sertoli cell cluster (Fig. 4C), suggesting functional Sertoli cells in ovotestis, which were consistent with the fact that Amh signaling is required for male development^47^. Further GO term enrichment showed that steroidogenesis process was enriched in Leydig cells. UMAP analysis of steroid biosynthesis indicated that male steroid biosynthesis factors, including *star*/*starl, cyp11a1, cyp17a1*, and *hsd3b1*, were highly expressed in Leydig cell cluster (Fig. 4D, E), which were key proteins for testosterone synthesis, In particular, Star and Starl were responsible for rate-limiting transport of cholesterol into the mitochondria for further steroidogenesis^48^, Cyp11a1, Cyp17a1, and Hsd3b1 worked in their downstream in the pathway (Fig. 4F). In addition, UMAP and immunofluorescence analysis showed that Sertoli and Leydig cells can be detected by Krt8, a protein for formation of intermediate filaments (Fig. 4G, H), and Sertoli and Leydig cells can also be detected by Cldn11, a tight junction protein (Fig. 4I, J). These results suggested that both Sertoli cells and Leydig cells were functional in ovotestis.

### Spermatogenesis arrests at round spermatid phase owing lack of transition from histone to protamine

**Fig. 5:**
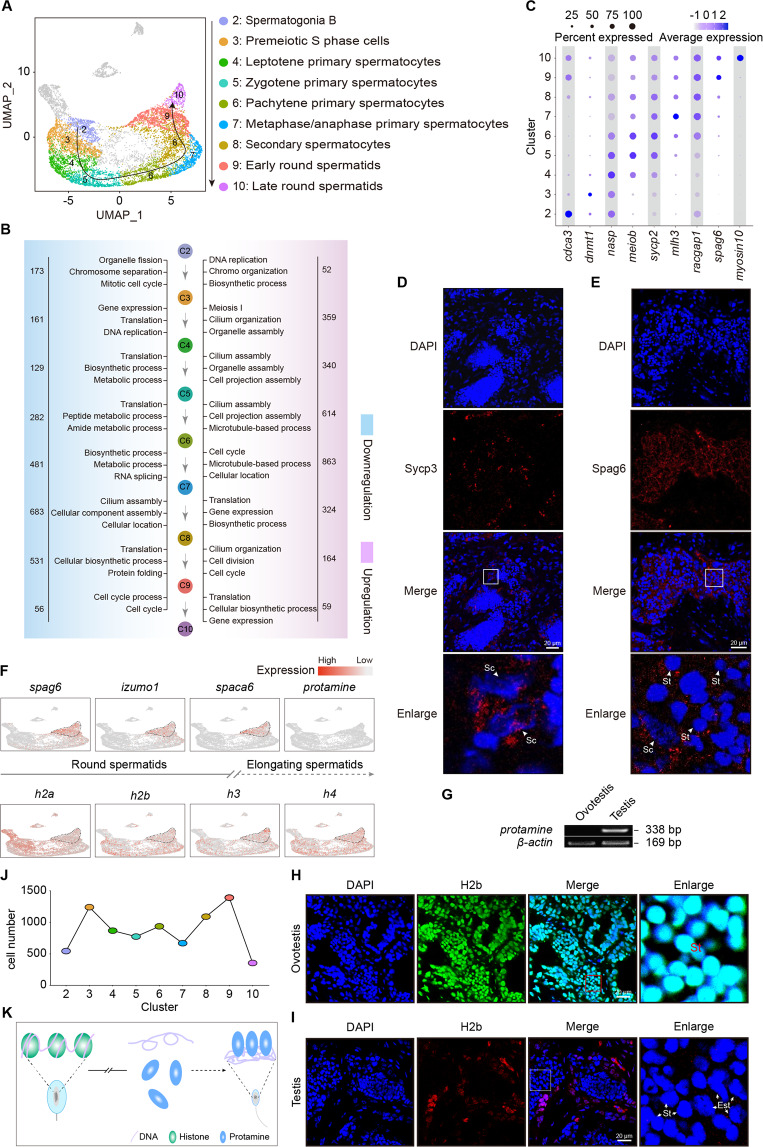
Spermatogenesis arrests at round spermatid phase owing to lack of transition from histones to protamine. **A** Developmental trajectory from spermatogonia B to round spermatids. **B** Representative gene ontology (GO) terms of upregulated and downregulated genes among clusters during spermatogenesis. Downregulated GO terms are in blue, upregulated GO terms are in pink. The number of upregulated and downregulated genes is indicated. **C** Dotplot showing the expression of representative genes in each cluster. **D** Immunofluorescence analysis of ovotestis using Sycp3 antibody (second antibody, TRITC-conjugated immunopure goat anti-rabbit IgG(H + L)). The nuclei were stained with DAPI. Sc spermatocytes. Scale bar: 20 µm. **E** Immunofluorescence analysis of ovotestis using Spag6 antibody (second antibody, TRITC-conjugated immunopure goat anti-rabbit IgG(H + L)). The nuclei were stained with DAPI. Sc spermatocytes, St round spermatids. Scale bar: 20 µm. **F** UMAP maps showing expression patterns of marker genes during spermatogenesis. Protamine was scarcely expressed, while histones were obviously expressed in round spermatids. **G** RT-PCR analysis of *protamine* expression pattern in ovotestis and testis. RT-PCR was performed using cDNAs as template. *β*-*actin* was used as an internal control. **H** Immunofluorescence analysis of ovotestis using H2b antibody (second antibody, FITC-conjugated immunopure goat anti-rabbit IgG(H + L)). The nuclei were stained with DAPI. St, round spermatids. Scale bar: 20 µm. **I** Immunofluorescence analysis of testis using H2b antibody (second antibody, TRITC-conjugated immunopure goat anti-rabbit IgG(H + L)). The nuclei were stained with DAPI. Round spermatids (St) and elongate spermatids (Est) are indicated by arrows. Scale bar: 20 µm. **J** Statistical analysis of cell number showing differentiation arrest from early round spermatids (cluster 9) to late round spermatids (cluster 10). **K** Schematic model of differentiation arrest from early round spermatids to late round spermatids owing to lack of transition from histone to protamine.

Phenotypically, round spermatids, but not elongated spermatids, were observed in ovotestis (Fig. 1C). Interestingly, two states (early and late) of round spermatids were detected (Fig. 5A). Both the number of expressed genes and the number of UMIs in the late stage of round spermatids were the most abundant among all clusters (Fig. S1C, D), suggesting active gene expressions in the late stage of round spermatids in ovotestis. To better understand the mechanisms of spermatogenesis arrest in the late round spermatids in ovotestis, developmental trajectory was used to track developmental order of the cell populations among clusters. This analysis indicated that developmental trajectory from cluster 2 to 10 represented meiosis process from spermatogonia B, spermatocytes, to late stage of round spermatids (Fig. 5A). Focused analysis of primary spermatocytes reveals developmental progression of the meiosis I, in which four subclusters, premeiotic S phase, leptotene/zygotene, pachytene, and metaphase/anaphase, were detected (Fig. S4A, B). Pseudotime trajectory with corresponding marker genes based on DEGs of each subcluster confirmed the developmental process of primary spermatocytes (Fig. S4C–G).

Gene ontology (GO) of both upregulated and downregulated genes among clusters during spermatogenesis showed functional changes with feature genes of high expression from spermatogonia B to round spermatids (Fig. 5B, C). Immunofluorescence analysis indicated expression of Sycp3, a marker of synaptonemal complex in spermatocytes, and Spag6, a component of the central apparatus, in round spermatids, suggesting normal development in spermatocytes and round spermatids (Fig. 5D, E). These analyses indicated that spermatogenesis processes from spermatogonia B, spermatocytes, to early stage of round spermatids proceeded properly. To get insight into spermiogenesis arrests, we performed expression analysis by UMAP mapping. This analysis indicated that representative genes of round spermatids, including *spag6*, *izumo1* and *spaca6*, were expressed in both cluster 9 and 10. In contrast, protamine was scarcely detected in both stages of round spermatids and even not in the whole ovotestis, but in testis (Fig. 5F, G). However, four types of histone genes, including *h2a, h2b, h3*, and *h4*, were expressed (Fig. 5F), and further immunofluorescence of H2b protein confirmed its expression in both stages of round spermatids in ovotestis (Fig. 5H), suggesting a histone retention in round spermatids in ovotestis. In contrast, in males, histone H2b was not observed in both round and elongate spermatids, suggesting that histones had been replaced by protamine in testis (Fig. 5I). Histone-to-protamine replacement is essential for producing functional sperm cells, and lack of histone replacement by protamine during post-meiotic transition of round spermatid to elongate spermatids led to male infertility^49^. Considering an enrichment of early round spermatids and a dramatic decline of late round spermatids (Fig. 5J), spermatogenesis would be arrested at early phase of round spermatids owing to lack of transition from histone to protamine (Fig. 5K). Overall, these data suggested that histone-to-protamine replacement cannot be performed due to deficiency of protamine expression, which thus led to developmental defects of elongate spermatids in ovotestis.

## Discussion

Infertile intersex often appears in individuals under certain pathological and physiological conditions, in which the gonad consists of both testis and ovary tissues, that is ovotestis. Patients who suffer from ovotestis are infertile and have many health problems^1^. However, how the ovotestis is formed remains elusive. Here, our study provides an overview of cell identities and a roadmap of germline, niche, and stem cell development in ovotestis, and uncovers the molecular and cellular mechanisms underlying ovotestis formation.

We found a common progenitor of germline stem cells with two developmental states, E-GSC and L-GSC, in ovotestis, which have a bipotential nature to differentiate into both spermatogonial stem cells and female germline stem cells. The developmental trajectories of five subtypes of germline stem cells confirmed the fate of differentiation to either oogenesis or spermatogenesis. Thus, E-GSC and L-GSC are similar to primordial germ cells (PGCs) in the developing embryonic gonad. The fact that they express a group of PGC markers, including *nanos2, nanos3*, *nanog*, and *dnd1*, also supports this viewpoint. Human PGCs are induced in the epiblast in early embryos and migrate to colonize the genital ridge in both sexes^50^. In male embryos, these PGCs can differentiate into spermatogonial stem cells and relevant germline cells, whereas in female embryos, they will develop into female germline stem cells and female germline cells^51^. In human adults, PGCs were not detected, and only PGC-like cells were observed in neonatal testes^52^. The findings of the common progenitors of germline stem cells, the E-GSC and L-GSC, in ovotestis, suggested that ovotestis possesses a developmental bipotential, attributed to the common stem cell progenitors. Our discovery of the common germline stem cell progenitors enriches our understanding of stem cell development.

Importantly, we uncovered developmental changes in the germline and niche cell lineages, and the molecular and cellular mechanisms underlying ovotestis formation. The mechanisms are involved in two disrupted processes of coordinating action. Female germline cells are degraded by nuclear phase separation of the proteasomes, and histone-to-protamine replacement is impaired in spermatid differentiation, which lead to developmental defects in both oogenesis and spermatogenesis processes in the ovotestis. On the one side, histone replacement by protamine is an essential process for chromatin remodeling in spermatid differentiation. Protamine mutations or aberrant histone retention results in failure of histone-to-protamine replacement, thus causing male infertility in humans and mice^49,53^. Our study reveals that deficiency of protamine expression is a major cause of infertility owing to lack of histone to protamine exchange in the round spermatid differentiation in ovotestis. On the other side, we found that nuclear phase separation of the proteasomes is a robust process in female germline cells, which is a degradation mechanism of ubiquitination-dependent proteasomes in the nuclei. Ubiquitination-mediated proteasome foci in the nucleoplasm were recently found in mammalian cells under various types of stress, including mouse stem cells, which can degrade key components in the nucleus, such as ribosomal proteins^42^. Consistent with this, loss-of-function mutations in most ribosomal proteins were lethal^43^. In addition, degradation of female germline cells is probably induced by aberrant signaling of the gonadal niche cells, owing to functional loss of the gonadal niche for oogenesis in the ovotestis. Thus, we suggest that the female germline cell degradation could be mediated by the nuclear proteasome pathway, together with deficiency signaling of the gonadal niche in ovotestis. Overall, our results provide a roadmap of germline, niche, and stem cell development in ovotestis and the corresponding molecular and cellular mechanisms underlying infertility.

## Materials and methods

### Experimental animals

The teleost fish *Monopterus albus* individuals were obtained from fish farms in Wuhan, Hubei Province, China. Adult animals (male, intersex) were used without randomization. All animal experiments and methods were performed in accordance with the relevant approved guidelines and regulations, as well as under the approval of the Ethics Committee of Wuhan University.

### Antibodies

Primary antibodies: Anti-Spag6 (A3088), Anti-Krt8 (A1024), Anti-Psmb2 (A5483), Anti-Cldn11 (A2593) were purchased from ABclonal, Wuhan, China. Anti-H2b (ET1612-25) was purchased from Huaan Biotechnology, Hangzhou, China. Anti-Sycp3 (ab97672) was purchased from Abcam, Cambridge, UK. Fluorescent antibodies: TRITC-conjugated ImmunoPure goat anti-rabbit IgG(H + L) (Cat# ZF-0316) and FITC-conjugated ImmunoPure goat anti-rabbit IgG(H + L) (Cat# ZF-0311) were purchased from Feiyi Technology, Wuhan, China.

### Plasmid constructs

Full-length *esr2b* (XM_020611557.1) was cloned into pcDNA3.0 using EcoRI and SalI to generate pCMV-*esr2b*. Three deletion fragments of the *atg13* (XM_020600753.1) promoter were amplified from genomic DNA, using KpnI and XhoI to generate the pGL3-basic vector (E1751, Promega, Madison, WI, USA). Full-length *rad23ba* (XM_020600944.1) was cloned into pSico-Cherry-FLAG using EcoRI and XhoI to generate Cherry-FLAG-*rad23ba*. Full-length *Rad23b* (NM_009011.4) was cloned into pSico-Cherry-FLAG using EcoRI and XhoI to generate Cherry-FLAG- *Rad23b*. Full-length *Psmb2* (NM_011970.4) and *psmb2* (XM_020597689.1) were cloned into pEGFP-N1 using XhoI and EcoRI to generate GFP-*Psmb2* and GFP-*psmb2*. The primers and PCR conditions are described in Table S4. Site-directed mutagenesis for the Esr2b binding sites was performed using the primers described in Table S4. All constructs were confirmed by sequencing (TSINGKE, Beijing, China).

### Cell culture and transfection

HEK293T cells (3142C0001000001715) and CHO cells (GDC0018) were obtained from China Center for Type Culture Collection. HEK293T cells were cultured in high glucose Dulbecco’s modified Eagle’s medium (DMEM) (SH30022.01B, HyClone, Logan, USA) with 12% fetal bovine serum (FBS) (P30-330250, PAN-Biotech, Aidenbach, Germany) and CHO cells were cultured in DMEM/F12 with 10% FBS. All cells were cultured at 37 °C in a 5% CO_2_ cell incubator. LipofectamineTM 2000 (11668027, Invitrogen, Shanghai, China) was used for transfection according to the routine protocol. For hyperosmotic stress, CHO cells were treated with 50 μM MG-132 (HY-13259, MCE, Shanghai, China) for 1 h, and then were stimulated using 0.2 M sucrose for 30 min (S818049, MACLIN, Shanghai, China).

### Immunofluorescence and living cell analysis

Immunofluorescence analysis was performed as described previously^54^. Briefly, CHO cells were cultured on glass coverslips, and gonadal tissues were embedded in OCT medium (4583, Tissue-Tek, Miles, USA) and cut into a series of 7 μm sections using a cryostat (CM1850, Leica, Germany). All samples were fixed with 4% paraformaldehyde for 20 min at room temperature and permeabilized with 1% Triton X-100 (9002-93-1, Sigma-Aldrich, USA) in PBS for 10 min and then blocked in 5% BSA for 30 min at room temperature. The sections were incubated with primary antibody in 5% BSA overnight at 4 °C. After being washed three times with PBS, the samples were subjected to the appropriate fluorescein-conjugated secondary antibody at 37 °C for 3 h. The nuclei were stained by DAPI (1155MG010, BioFroxx, Germany). Images were captured by confocal fluorescence microscopy (SP8, Leica, Germany). For time-lapse experiments, CHO cells were initially plated in 35 mm glass-bottomed dishes (BS-20-GJM, Biosharp, Hefei, China). The cells were transfected with pEGFP-*psmb2* plasmid when the cell density reached 50–60%. 4 h after transfection, the medium was replaced by fresh medium. After 24 h, the medium was discarded, cells were washed using PBS for one time, treated with 0.2 M sucrose for 30 min, and imaged for 1–2 h. Finally, images were captured by Zeiss confocal fluorescence microscopy (LSM880, Zeiss, Oberkochen, Germany) with an incubator (humidified environment at 37 °C under 5% CO_2_). The data were processed by ZEN software (Zeiss, Oberkochen).

### Dual-luciferase reporter assays

For luciferase assays, 293 T cells per well (48 well plate) were transfected with 0.5 μg recombinant constructs and 1 ng pRL-TK (E2241, Promega). Then luciferase activities were measured by a dual-luciferase reporter assay system (E1980, Promega) and a Modulus Single Tube Multimode Reader (Turner Biosystems, Sunnyvale, CA, USA) according to the manufacturer’s protocol. The experiments were repeated at least three times, and the results were expressed as the means ± SD.

### Semiquantitative RT-PCR

TRIzol (R401-01-AA, Vazyme, Nanjing, China) was used to isolate total RNA, which was reverse transcribed using a poly (T)18 primer and MMLV reverse transcriptase (M1701, Promega). Semiquantitative RT-PCR was used to amplify cDNAs from ovotestis and testis tissues. *β-actin* was used as an internal control. The primer sequences are described in Table S4.

### Ovotestis sample preparation for single-cell RNA sequencing

Gonads were collected and washed twice in 1xPBS. Sex was verified by histological analysis of the gonad sections. The sections were stained by hematoxylin and eosin and images were captured using a Leica microsystem (Leica). Three samples were minced with sterilized scissors after washing three times with D-Hank’s, then were digested in collagenase type IV (C5138, Sigma-Aldrich, St Louis, USA) of 1 mg/ml at 28 °C for 20 min, with shaking up and down once in every 3 min. The tubes were spun at 300g and the supernatant was removed. The samples were washed three times with D-Hank’s, then digested with 0.25% trypsin (1541894, gibco, Grand Island, NY, USA) and Deoxyribonuclease I (EN0521, Thermo Scientific, Rockford, IL, USA) buffered by Leiboviz’s L-15 (SH30525.01, HyClone, Utah, USA) medium for 10 min, with shaking up and down once in every 2 min. The digestion was stopped with Leiboviz’s L-15 medium supplemented with 10% FBS (P30-330250, PAN-Biotech, Aidenbach, Germany). After filtering by strainers with mesh sizes of 70 μm (15-1070, Biologix, Jinan, Shandong Province, China) and 40 μm (15-1040, Biologix), single cells were obtained. The cells were pelleted by centrifugation at 300g for 5 min and resuspended with Leiboviz’s L-15 medium supplemented with 10% FBS. The cell viability was evaluated by trypan blue (T6146, Sigma–Aldrich) staining with a hemocytometer.

### Library preparation and sequencing

Chromium single-cell 3′ reagent kits V3 (10X Genomics, Pleasanton, CA, USA) were used for scRNA-seq library preparation according to the manufacturer’s protocols. A single-cell suspension was loaded into the Chromium Controller (10X Genomics) to form single cell GEMs (Gel Bead-in-Emulsions), which were droplets including a bead with cell barcode and a cell in every droplet. RT-PCR reaction was performed in the individual GEM and synthetic cDNA with a specific cell barcode was mixed for amplification; then the cDNA library was prepared based on the manufacturer’s instructions (10X Genomics). Sequencing was performed on a BGISEQ-500 (BGI, Shenzhen, China).

### Mapping and processing of single-cell RNA-seq data

Raw sequencing data were demultiplexed using the mkfastq application of CellRanger (CellRanger v 3.1.0) with default parameters to get Fastq files. Reads were aligned to reference genome (M_albus_1.0) using STAR^55^, and barcode and UMI counting were performed to generate feature-barcode matrices by CellRanger count application (Cell Ranger v 3.1.0) with default settings. The feature-barcode matrices were used for further analysis.

Using Seurat package (v3.1.0)^56^ with default parameters, we checked the single cell data to filter cells of good quality. The cells were retained when the expressed gene number of cells was more than 200 and the maximum expressed gene number was less than 90%, with less than 5% of mitochondrial gene expression. We normalized gene expression values relative to the total number of reads in the cell, and the resulting expression values were then multiplied and log-transformed. Subsequently, the most highly variable genes of 2000 were used for further clustering analysis. Nonlinear dimensionality reduction was performed by principal component analysis and a graph-based clustering approach was used for cell clustering. Then, two-dimensional visualization of clusters was generated by uniform manifold approximation and projection (UMAP), which is a nonlinear dimensionality-reduction technique^35^. For each cluster, differentially expressed genes were identified by FindConservedMarkers application (logfc. threshold >0.25 and minPct >0.25) of Seurat. Cell types were identified and annotated manually based on the previously known markers and the cluster specific genes. Pseudotime trajectory analysis was performed by Monocle (v2.10.1)^57^ with default settings based on Seurat clustering data sets.

### Gene functional annotation

GO term and KEGG pathway enrichment analysis of DEGs were performed with online software g:Profiler^46^, which supported statistical analysis and visualization. Representatively significant biology processes and KEGG pathways were selected for further studies, and enrichments with *P*-adjusted < 0.05 were considered as significant. Open reading frames of DEGs were recognized by GETORF (EMBOSS: 6.5.7.0)^58^, then the ORFs were aligned to the AnimalTFDB database^59^ to annotate transcription factors of DEGs using DIAMOND (v0.8.31)^60^. Transcription factor binding sites were predicted using JASPAR online software^61^. *P*-adjusted of all used DEGs were less than 0.05.

## Supplementary information

Supplementary information

Supplementary figure S1

Supplementary figure S2

Supplementary figure S3

Supplementary figure S4

Time-lapse imaging of the proteasome foci fusion 1 in nucleus

Time-lapse imaging of the proteasome foci fusion 2 in nucleus

## Data Availability

The accession number for the sequence reported in this paper is GEO: GSE153961.
