# Supplementary material for: Cellular fate of intersex differentiation
Source: Cell Death Dis. 2021 Apr 12;12(4):388. doi: 10.1038/s41419-021-03676-x (PMC8041806; doi:10.1038/s41419-021-03676-x)
Supplement: Supplementary file 1 — Supplementary information [file 41419_2021_3676_MOESM1_ESM.docx]

**Supplementary information**

**Figure S1.** **Identification of major cell types in ovotestis at single-cell level.** (A) Uniform Manifold Approximation and Projection (UMAP) plot revealing 13 cell clusters in ovotestis (left panel), dotplot exhibiting expression of representative genes in spermatocytes at different stages of differentiation (right panel). (B) UMAP maps showing expression levels of marker genes in different cell populations. Clusters of specific genes are highlighted by black circles. (C) Boxplot indicating the number of expressed genes in each cluster. (D) Boxplot showing the number of unique molecular identifiers (UMIs) in each cluster.

**Figure S2. Number change of upregulated and downregulated genes in the trajectories 1 and 2, and relative expressions of representative genes for DNA synthesis and mitosis in subclusters 1-5, related to Figure 1**. (A) Histogram showing number change of upregulated and downregulated genes in the trajectories 1 and 2 respectively. (B) Relative expressions of representative genes (*gmnn*, *mcm2*, *rfc4*, *rpa1*, *ssbp1*) for DNA synthesis among subclusters. (C) Relative expressions of representative genes (*aspm*, *chtf8*, *nsl1*, *tpx2*, *kif20a*) for mitosis among subclusters.

**Figure S3.** **Apoptosis, autophagy and nuclear proteasomes involved in degradation of female germline cells, related to Figure 2.** (A) UMAP map showing cells in subcluster 3 (red). (B) Dotplot showing the expression of apoptosis genes among subclusters. (C) Dotplot revealing the expression patterns of autophagy genes at different stages of autophagosome formation and fusion with lysosome among subclusters. (D) Dotplot indicating the expression patterns of key genes *atg13* and *esr2b*. (E) Schematic showing the position of Esr2b binding sites. Top panel showing sequence logo of Esr2b binding site predicted by JASPAR database, and bottom panel showing the position of Esr2b binding site on *atg13* promoter region. (F) Luciferase assay showing the activities of a series of deleted constructs in 293T cells. The mean ± SD were from 3 independent experiments, **P < 0.01. (G) Point mutation analysis of the promoter using luciferase assays in 293T cells. Top panel showing the DNA sequences of *atg13* mutants (Mut1 and Mut2) and wild type. Bottom panel indicating luciferase assays of point mutation. Only Mut1 can lead to an obvious decrease of luciferase activity. The mean ± SD were from 3 independent experiments, *P < 0.05; **P < 0.01. (H) Overexpression of *esr2b* up-regulated promoter activities of *atg13* in 293T cells. The mean ± SD were from 3 independent experiments, *P < 0.05; **P < 0.01 (I) Schematic representation of autophagic degradation mechanisms of female germline cells. (J) Co-localization of Psmb2 and Rps2 in nucleus. The cells were stimulated with 0.2 M sucrose for 30 min. Psmb2 (red) and Rps2 (green) were colocalized in the nuclei. The nuclei were stained by DAPI. Scale bar, 5 µm.

**Figure S4.** **Identification of the meiosis I in primary spermatocytes, related to Figure 5.** (A) UMAP map showing primary spermatocyte clusters (coloured). (B) Reclustering primary spermatocyte clusters into 4 subclusters: premeiotic S phase, leptotene/zygotene, pachytene, and metaphase/anaphase subcluster. (C) Pseudotime trajectory analysis of 4 subclusters by Monocle. (D) Expression pattern of meiosis genes and methylation genes along the pseudotime axis. (E) Dotplot indicating expression of representative genes in each subcluster. (F) Histogram showing number change of upregulated and downregulated genes in the trajectory. (G) Representative GO terms based on DEGs of each subcluster.

**Supplementary video 1**

**Time-lapse image 1 of the proteasome foci fusion in nucleus**. Time-lapse imaging showed that the proteasome foci with Psmb2 can fuse together, suggesting a nature of liquid droplets of the foci in the living cells.

**Supplementary video 2**

**Time-lapse image 2 of the proteasome foci fusion in nucleus**. Time-lapse imaging showed that the proteasome foci with Psmb2 can fuse together, suggesting a nature of liquid droplets of the foci in the living cells.

**Table S1. Basic data of single cell RNA sequencing**

| Number of reads | Mean reads per cell | Valid barcodes | Q30 bases in barcode | Q30 base in RNA | Q30 base in UMI |
| --- | --- | --- | --- | --- | --- |
| 474190611 | 47419 | 96.5% | 90.8% | 84.9% | 89.2% |

| Estimated number of cells | Mean reads per cell | Median genes per cell | Total genes detected | Median UMI counts per cell |
| --- | --- | --- | --- | --- |
| 10000 | 47419 | 3126 | 22184 | 9259 |

**Table S2. Gene expression information**

**Table S3. Gene information**

| Gene symbol | Gene ID | GenBank Accession No. |
| --- | --- | --- |
| *nanos3* | 109957874 | NW_018127909 |
| *nanos2* | 109971341 | NW_018128101 |
| *kif20a* | 109959520 | NW_018127919 |
| *kmt5a* | 109961594 | NW_018127936 |
| *zar1* | 109962058 | NW_018127941 |
| *zar1l* | 109971876 | NW_018128117 |
| *spo11* | 109966120 | NW_018127986 |
| *mns1*  *izumo1*  *spaca6*  *amh*  *wt1b*  *cyp17a1*  *star*  *bmp4*  *dcn*  *id1*  *smad7*  *cyp11a1*  *hsd3b1*  *starl*  *krt8*  *cdca3*  *dnmt1*  *nasp*  *sycp2*  *mlh3*  *racgap1*  *spag6*  *myosin10*  *protamine*  *h2a*  *h2b*  *h3*  *h4*  *sycp1*  *dmrt1*  *dnmt3aa*  *piwil2*  *rec8*  *sycp3* | 109963533  109956588  109964877  109973075  109969035  109973668  109954875  109968000  109959873  109956315  109960667  109971254  109951646  109963038  109970400  109966204  109965946  109961755  109969018  109955142  109970295  109965516  109972762  109956575  109972027  109952335  109953628  109954057  109971909  109951421  109962317  109954762  109968807  109969077 | NW_018127884  NW_018127900  NW_018127973  NW_018128159  NW_018128048  NW_018128179  NW_018127892  NW_018128021  NW_018127921  NW_018127898  NW_018127929  NW_018128098  NW_018128281  NW_018127949  NW_018128077  NW_018127987  NW_018127985  NW_018127938  NW_018127886  NW_018127893  NW_018128074  NW_018127980  NW_018127887  NW_018127900  NW_018128123  NW_018128334  NW_018128751  NW_018130726  NW_018127881  NW_018128265  NW_018127943  NW_018127892  NW_018128042  NW_018128050 |
| *pcna* | 109956536 | NW_018127900 |
| *gtf3ab*  *tfap4*  *spen*  *tpm1*  *figla*  *zglp1*  *smc1a*  *nme5*  *c9orf117*  *dnajb13*  *ttc25*  *e2f8* | 109960132  109972901  109967192  109966604  109954787  109958463  109969155  109973140  109951419  109959464  109964191  109967030 | NW_018127924  NW_018128153  NW_018128006  NW_018127885  NW_018127892  NW_018127912  NW_018127886  NW_018128162  NW_018128265  NW_018127919  NW_018127965  NW_018128003 |
| *cenpu*  *g2e3*  *mybl2b*  *e2f7*  *dnmt1*  *nsd2 fhl3a*  *nkx1-2*  *olig2*  *swap70a*  *znf207*  *chrnd*  *vim*  *npas4*  *snrnp27*  *snrpb*  *calr*  *alyref*  *top2a*  dnd1  *ybx2*  *nanog*  *foxl2*  *cyp19a1a*  *esr2b*  *rad23ba*  *rad23bb*  *atg13*  *ulk2*  *vmp1*  *pik3r4* | 109957543  109951733  109956152  109960970  109965946  109962432  109954715  109960516  109972462  109966099  109958421  109970934  109955479  109954575  109954870  109966897  109967433  109958939  109963627  109966434  109951390  109968784  109951921  109963333  109966606  109960675  109972195  109960566  109952850  109960373  109970105 | NW_018127907  NW_018128283  NW_018127898  NW_018127931  NW_018127985  NW_018127944  NW_018127891  NW_018127883  NW_018128137  NW_018127884  NW_018127912  NW_018128089  NW_018127894  NW_018127882  NW_018127892  NW_018128001  NW_018128009  NW_018127915  NW_018127956  NW_018127991  NW_018128264  NW_018128042  NW_018127888  NW_018127952  NW_018127995  NW_018127929  NW_018128130  NW_018127928  NW_018128385  NW_018127926  NW_018128069 |
| *becn1*  *atg5*  *atg7*  *atg2b*  *atg4b*  *map1lc3a*  *map1lc3b*  *wipi2*  *epg5*  *tecpr1b*  *lmna1*  *uba1*  *ube2a*  *ube2d2*  *ube2e1*  *ube2e2*  *ube2h*  *ube2i*  *ube2j2*  *ube2q2*  *cdc34a*  *eloca*  *fbxo4*  *fzr1b*  *vcp*  *gadd45a*  *bax*  *casp3a*  *casp3b*  *casp6*  *dffa*  *rad21*  *dmc1*  *spata22*  *hormad1*  *rad51*  *mcm2*  *psmd6*  *gmnn*  *rfc4*  *rpa1*  *ssbp1*  *chtf8*  *nsl1*  *tpx2*  *smo*  *ptch1*  *ptch2*  *hhip*  *nid1*  *gli1*  *gli2*  *gli3*  *psmb2* | 109963002  109968941  109963128  109955026  109965757  109966899  109962563  109972424  109956912  109969479  109974709  109969378  109958185  109957516  109963674  109965582  109959828  109963560  109956010  109973961  109964048  109960118  109956672  109970656  109956931  109969313  109964251  109972113  109957512  109955561  109969931  109966736  109971849  109960463  109968935  109966564  109952616  109955919  109952907  109952570  109968543  109964982  109974392  109951878  109951051  109959936  109956558  109957709  109955575  109960712  109963857  109963787  109970136  109958783 | NW_018127949  NW_018128046  NW_018127881  NW_018127893  NW_018127983  NW_018128001  NW_018127945  NW_018128136  NW_018127903  NW_018128054  NW_018128224  NW_018127886  NW_018127882  NW_018127907  NW_018127957  NW_018127981  NW_018127921  NW_018127956  NW_018127898  NW_018127887  NW_018127963  NW_018127924  NW_018127900  NW_018128084  NW_018127903  NW_018128052  NW_018127965  NW_018128127  NW_018127907  NW_018127895  NW_018127886  NW_018127998  NW_018128116  NW_018127927  NW_018128046  NW_018127994  NW_018128359  NW_018127898  NW_018128394  NW_018128355  NW_018128032  NW_018127974  NW_018128212  NW_018128297  NW_018128241  NW_018127921  NW_018127900  NW_018127908  NW_018127895  NW_018127883  NW_018127881  NW_018127959  NW_018128070  NW_018127914 |

**Table S4 Primer sequences and PCR conditions**

| Genes/fragments | Primer sequence (5’-3’) | Tm (^o^C) |
| --- | --- | --- |
| *protamine*-F  *protamine*-R  *β-actin*-F  *β-actin*-R  *rad23ba*-F  *rad23ba*-R  *Rad23b*-F  *Rad23b*-R  *Psmb2*-F  *Psmb2*-R  *atg13*-LS1-F  *atg13*-LS2-F  *atg13*-LS3-F  *atg13*-R  *atg13*-mut I-F  *atg13*-mut I-R  *atg13*-mut II-F  *atg13*-mut II-R  *psmb2-*F  *psmb2-*R  *rps2-*F  *rps2-*R  *rps26*-F  *rps26*-R | GGTTACGACGTGGTGAAGAAC  CGTTTAGCCTTCTTGGGACTC  TCTTCCAGCCTTCTTTCCTTG  ATCTCCTTCTGCATCCTGTCA  AATGAATTCATGCAGATAACTTTGAAAACT  AATCTCGAGTCAATCATCGTCAAAGTTCTG  AATGAATTCATGCAGGTCACCCTGAA  AATCTCGAGTCAGTCTTCATCAAAGTTCTG  AATCTCGAGATGGAGTACCTCATTGGCATC  AATGAATTCCGGAGTCCCGCTTAGGGAAGGC  AATGGTACCTTAGCTGAAGTGTTTGAGTAT  AA GGTACCGTGCTGTTTCCCTTTTTTTAT  AATGGTACCAGCATACATTCGCAGATTCGA  AATCTCGAGACTTTCGTAGATGTTTTGTTT  AAAAAAATATCTTGCAGCATACATTCGCAGAT  AAAAAAAGTAGTTTGTTTTGGTTGTCAAATGC  AAAAAAATGCTAGCTAGCTAGCTACTTGGTGT  AAAAAAAGTTCCATTTATACAGTAATAAGTAA  AATCTCGAGATGGAATATTTGATCGGGATC  AATGAATTCCCTTGGCGCCATGGG  AATCTCGAG ATGGCGGACGACGCCGGT  AATGAATTCCGGAGGTAGCTGCCTGGACAG  AATCTCGAGATGACCAAGAAGAGGAGGAA  AATGAATTCCCATAGGCTTTGGAGGAGCTC | 58  58  57  55  57  60  60  55  55  63  57  60 |

Underlined sequences (AAAAAAA) in *atg13* were mutated from wild type sequences (site 1, AGGTCAGGAACACC and site 2, AGGTCGGCCCGACA)
