# Supplementary figures and images for: Cellular fate of intersex differentiation
Source: Cell Death Dis. 2021 Apr 12;12(4):388. doi: 10.1038/s41419-021-03676-x (PMC8041806; doi:10.1038/s41419-021-03676-x)

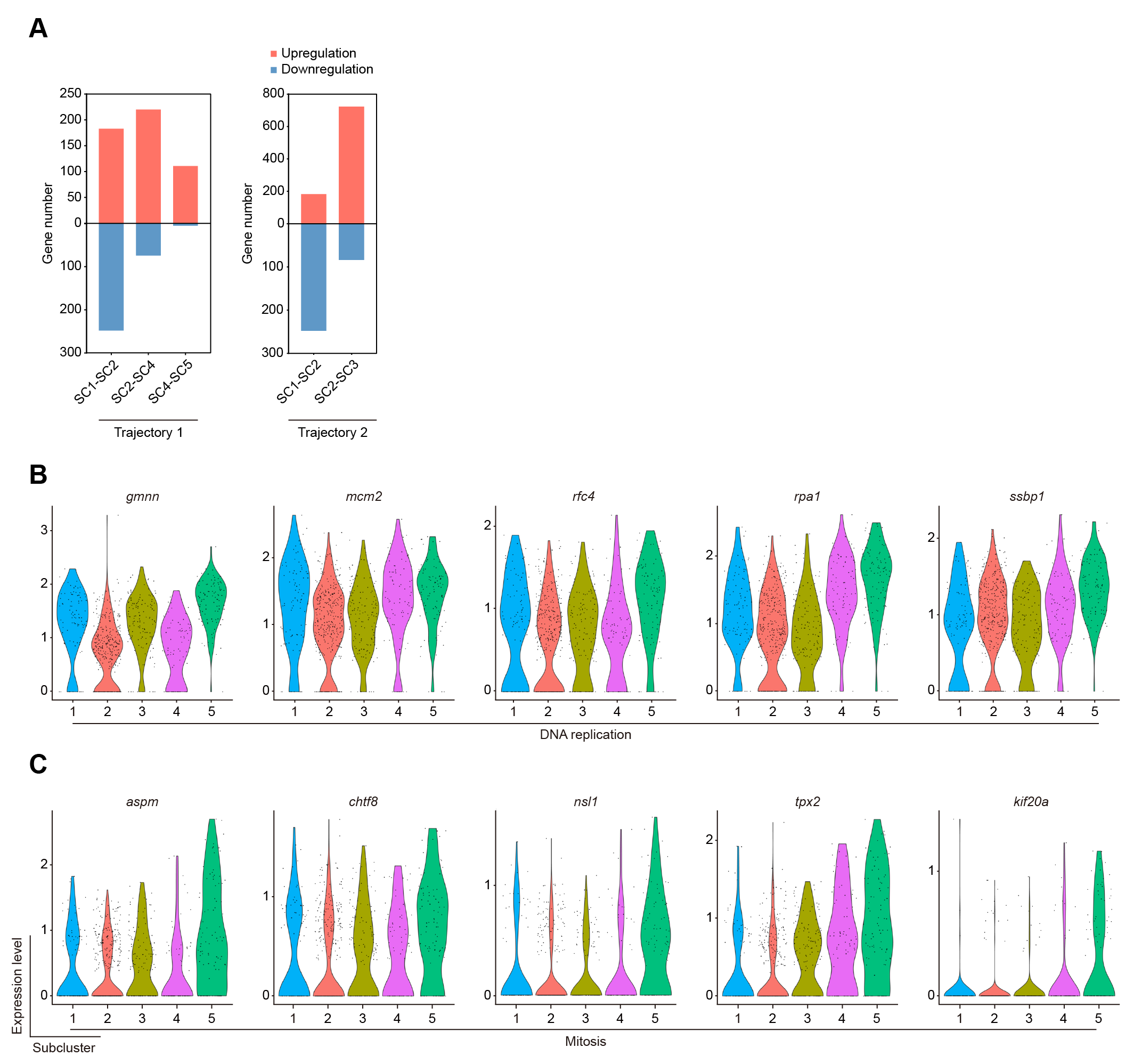

Supplement: Supplementary file 3 — Supplementary figure S2 [file 41419_2021_3676_MOESM3_ESM.png]

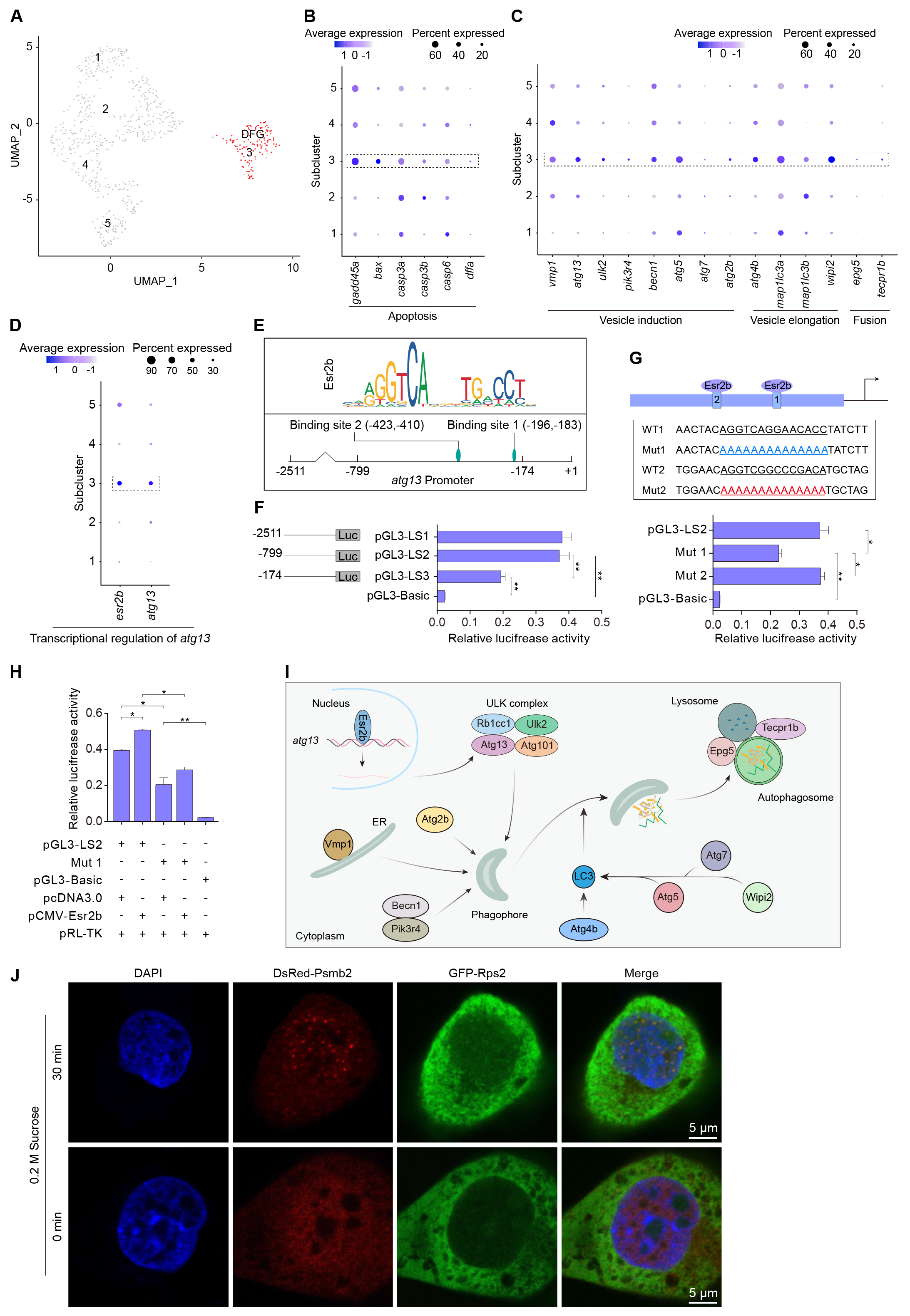

Supplement: Supplementary file 4 — Supplementary figure S3 [file 41419_2021_3676_MOESM4_ESM.png]

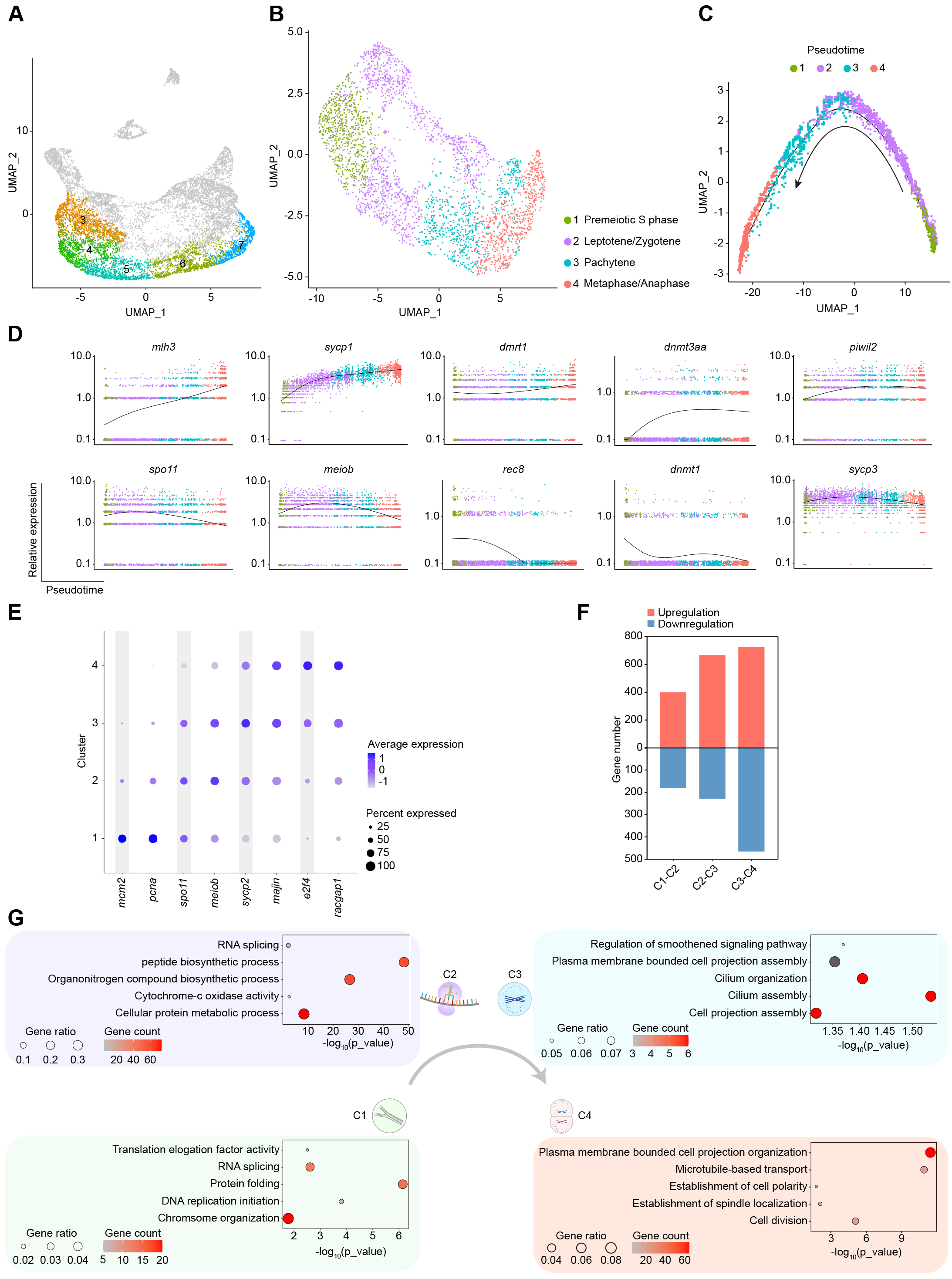

Supplement: Supplementary file 5 — Supplementary figure S4 [file 41419_2021_3676_MOESM5_ESM.png]
